# Supplementary material for: Enhancing the Antimicrobial Properties of Peptides through Cell-Penetrating Peptide Conjugation: A Comprehensive Assessment
Source: Int J Mol Sci. 2023 Nov 24;24(23):16723. doi: 10.3390/ijms242316723 (PMC10706425; doi:10.3390/ijms242316723)
Supplement: Supplementary file 1 [file ijms-24-16723-s001.zip › Supplementary File S3.pdf]

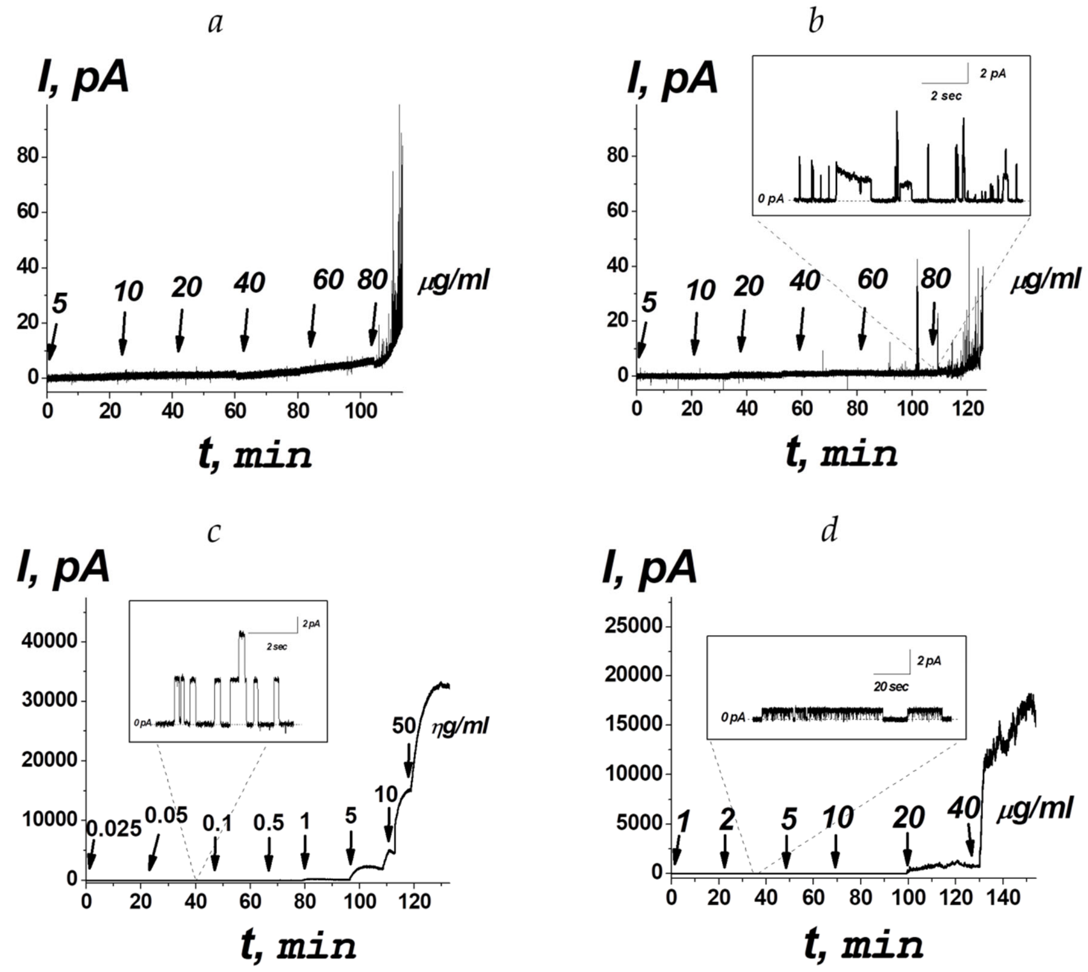

Supporting Figure S1. Typical time courses of the transmembrane current flowing through the lipid bilayers after addition of detergent Triton X-100 (**a**), antimicrobial peptide from *Xenopus laevis* magainin I (**b**), antimicrobial peptide from *Bacillus brevis* gramicidin A (**c**), and lantibiotic from *Streptoverticillium cinnamoneus* duramycin (**d**). The membranes were composed of POPE and POPG (50:50 mol%) and bathed in 0.1 M KCl, pH 7.4. The transmembrane voltage was equal to 100 mV. The moments of detergent or peptide addition are indicated by arrows. The corresponding concentrations of the membrane modifying agents in the membrane-bathing solution are shown above the arrows. *Insets*: examples of current tracks presenting openings and closures of single ion-permeable pores induced by magainin I (**b**), gramicidin A (**c**), and duramycin (**d**).
