# Supplementary material for: Enhancing the Antimicrobial Properties of Peptides through Cell-Penetrating Peptide Conjugation: A Comprehensive Assessment
Source: Int J Mol Sci. 2023 Nov 24;24(23):16723. doi: 10.3390/ijms242316723 (PMC10706425; doi:10.3390/ijms242316723)
Supplement: Supplementary file 1 [file ijms-24-16723-s001.zip › Supplementary File S4.pdf]

Table S1. Antibacterial activity for amyloidogenic fragment ITDFGIFIGL (I10L) from S1 *P. aeruginosa*.

	0.9 μM	9.1 μM	91.3 μM	913 μM	9130 μM
B. cereus	-	-	-	+	+
MRSA	-	-	-	+	+
S. aureus	-	-	-	+	+
E. coli	-	-	-	+	+
P. carotovorum	-	-	-	+	+
